# Dai Huang Fu Zi Tang could ameliorate intestinal injury in a rat model of hemorrhagic shock by regulating intestinal blood flow and intestinal expression of p-VASP and ZO-1

**DOI:** 10.1186/1472-6882-14-80

**Published:** 2014-03-01

**Authors:** Xiaoguang Lu, Xin Kang, Libin Zhan, Chunyu Lv, Zhiwei Fan, Yingli Wang, Robbie Ali, Chang Lv, Siyao Li, Jinhai Mu

**Affiliations:** 1 Department of Emergency Medicine, Zhongshan Hospital, Dalian University, Dalian 116001, China; 2 College (Institute) of Integrative Medicine, Dalian Medical University, Dalian 116044, China; 3 Department of Traditional Chinese Medicine, the Second Affiliated Hospital, Dalian Medical University, Dalian 116023, China; 4 Graduate School of Zunyi Medical University, Zunyi 563003, China; 5 Windber Hospital Inc, Windber Medical Center, Windber, PA 15963, USA; 6 Graduate School of Dalian University, Dalian 116044, China

**Keywords:** Hemorrhagic shock, Dai Huang Fu Zi Tang, Endotoxin, Small intestine, Phosphorylated vasodilator-stimulated phosphoprotein (p-VASP), Zonula occludens (ZO)-1 protein, Intestinal fatty acid binding protein (IFABP)

## Abstract

**Background:**

Dai Huang Fu Zi Tang (DHFZT), an oriental herbal formula, has long been used clinically in treatment of intestinal obstruction, acute pancreatitis, cholecystalgia and chronic diarrhea for thousands of years. Recent studies have demonstrated that DHFZT can reduce intestinal pathological injury and the concentration of enterogenous endotoxin in an animal model. But the underlying mechanism has not been fully elucidated.

**Methods:**

SD male rats in adult were used to model HS and treated with DHFZT. The serum concentration of endotoxin were analyzed by dynamic turbidimetric method. In addition, the blood flow of small intestine were measured using laser speckle technique. Phosphorylated vasodilator-stimulated phosphoprotein (p-VASP) and zonula occludens (ZO)-1 protein, intestinal fatty acid binding protein (IFABP) were measured by Western Blotting, RT-PCR, ELISA respectively.

**Results:**

Present study showed that DHFZT markedly elevated the blood flow of small intestine, protected the intestinal barrier function by up-regulating the expression of ZO-1 protein and down-regulating expression of p-VASP, and notely decreased serum concentration of IFABP and endotoxin in HS.

**Conclusions:**

These results reveal that DHFZT improves intestinal blood flow, protects the intestinal barrier function, and ameliorates intestinal endotoxaemia in rats with HS.

## Background

Hemorrhagic shock (HS) accounts for 30% of deaths from injury around the world in 2008. Its mortality range 36.5% from 69% [1,2]. HS is a clinical syndrome resulting from circulatory dysfunction that leads to decreased tissue ischemia, the accumulation of oxygen debt, and ultimately to multiple organ injury and death if left untreated [3]. Many previous studies have shown that after HS, intestinal ischemia continuously and Gut-derived endotoxemia are very important driving factor of HS into multiple organ dysfunction syndrome (MODS) [4-6]. So, how to increase the intestinal blood flow, protect intestinal normal function and reduce the serum endotoxin level are the key steps of improving the prognosis and reducing mortality.

Dai Huang Fu Zi Tang(DHFZT), a prescription in traditional Chinese medicine (TCM), has been used to cure Acute appendicitis, acute intestinal obstruction, acute pancreatitis, shock, etc [7-10]. DHFZT composed of three herbs including *Radix et Rhizoma Rhei (DH)*, *Radix Aconiti Lateralis Praeparata (FZ)* and *Radix et Rhizoma Asari (XX)*, was originally described in the Synopsis of Golden Chamber (Jin Kui Yao Lue), a treatise on febrile and miscellaneous diseases written by the outstanding physician Zhang Zhongjing in Han Dynasty.

Our preliminary experimental results demonstrated that DHFZT had reduced serum concentration of endotoxin and protected the intestinal barrier function of rats [11]. However,the underlying mechanisms of these protective effects remain unclear. In this study, we applied a HS model to discuss mechamism of DHFZT in rats.

## Methods

### Material

Rat intestinal fatty acid binding protein (IFABP) ELISA kit was purchased from Cusabio Co., Ltd (Wuhai, China). EKT-5 M set dynamic Gram-negative bacteria test kit was obtained from Gold Mountainriver Techn Development Co. Ltd (Beijing, China). Rabbit anti-rat phosphorylated vasodilator-stimulated phosphoprotein (p-VASP) antibody was purchased from Cell Signaling Technology, Inc (Boston, United States). Rabbit anti-rat zonula occluden (ZO-1) antibody was obtained from Santa Cruz Biotechnology, Inc (California, United States). SP9001 anti-rabbit immunohistochemical detection kit and DAB reagent kit were purchased from Beijing Zhongshan Golden Bridge Biotechnology Co., Ltd (Beijing, China).

### Preparation and quality controls of DHFZT

DHFZT is composed of 3 species of herbal plants,each dried crude drug of which were purchased from Tong Ren Tang Group Co., Ltd (Beijing, China). The formula of DHFZT is described in Table 1, and voucher specimen of *Rheum palmatum Linn* (No.00000022), *Aconitum carmichaeli Debeaux* (No.01814237), and *Asarum heterotropoides F.S chmidt var.mandshuricum* (No.00916696) are kept in Institute of Botany, the Chinese Academy of Sciences. The herbal components were identified by one of the authors [12]. To keep the consistency of the herbal chemical ingredients, all of the herbal components were originally obtained from the standard native sources as stated above with GAP grade and the drugs were extracted with standard methods according to Chinese Pharmacopeoia III (edition 2010). Standard substance, such as *Rheum emodin*, *rhein*, *rhubarb phenol*, *aconitine and physcion*, with purity of 99% or higher were purchased from the National Institute for the Control of Pharmaceutical and Biological Products (Beijing, China). *Methyl eugenol* and *Asarum ether* were purchased from Sigma, St (Louis, Mo, United States). Its chemical ingredients were confirmed at Chemical Analysis Center of Technology Institute, Dalian University of Technology.

**Table 1:** Herbal compositions of DHFZT

**Scientific name**	**Herbal name**	**Quantity (dry, g)**
Rheum palmatum Linn	Radix et Rhizoma Rhei (DH)	9.0
Aconitum carmichaeli Debeaux	Radix Aconiti Lateralis Praeparata (FZ)	9.0
Asarum heterotropoides F. Schmidt var. mandshuricum	Radix et Rhizoma Asari (XX)	3.0
Total		21.0

According to the original prescription from the 〝Jin Kui Yao Lue〞, DH, FZ and XX were mixed in the ration of 3:3:1 (w/w). First, FZ were soaked in water (1:25) for 30 min, followed by extraction in boiling water (100°C) for 1 h. Then DH was added and boiled for 10 min. Finally, XX was added and boiled for 5 min. The DHFZT were concentrated by rotary evaporator (Heidolph Instruments, Germany) and lyophilized to obtain dry extract through freeze-drying system (Labconco, United States) at −80°C,yielding final 3.72 g (extraction ratio 17.71%), and stored at 4°C for use. The lyophilized DHFZT extract was dissolved in an appropriate volume of 0.9% normal saline prior to administrating to rats.

### Animals

A total of 72 Sprague–Dawley male rats (age, 5–7 weeks; weight, 250–300 g) were purchased from the Dalian Medical University Experimental Animal Center. Animals were housed in a colony room under a 12:12 light–dark cycle (lights on from 7:30 a.m. to 7:30 p.m.) at constant temperature (23 ± 1°C) and humidity (50 ± 5%). All procedures involving animals were conducted in conformity with the National Institute of Health Guide for the Care and Use of Laboratory Animals and were approved by the Dalian University Animal Research Ethics Committee. Anesthetic drugs and all other necessary measures were used to reduce animal suffering during experimental procedures.

### Experiments design

The experiment aimed to test whether DHFZT could increase intestinal blood flow, and protect barrier function of intestine, and then decrease intestinal endotoxaemia in rats with HS. Rats were anesthetized with ether inhalation for narcotic induction and then by injecting 2% of sodium pentobarbital (40 mg/kg) into the abdominal cavity for maintaining anesthesia. Morphine hydrochloride (5 ~ 10 mg/kg) by subcutaneous injection was used to ease pain 20 min before anesthesia. With aseptic technique, poly-ethylene (PE50) catheters were placed in the right carotid artery for continuous mean arterial pressure (MAP) monitoring with a multifunctional physiological recorder (BIOPAC, USA) in the left femoral artery for blood withdrawal and in the right femoral vein for fluid infusion. A rat model with controlled HS model was induced following a simple modification of the method described previously [13,14]. The MAP was gradually reduced to 40 mmHg by withdrawing blood within 10 min in a syringe prerinsed with 0.02 ml heparin (1,000 IU/ml), and stopped bloodletting and then maintained around 40 mmHg for 60 min before fluid resuscitation was initiated. HS model of rat was thus successfully developed, followed by resuscitation with all the blood released and 2 times amount of normal saline via the right femoral vein. In addition, Shock Index (SI) was used to assess whether HS model was succesful or not. SI is less than 0.5, which shows that the body is no shock. SI is greater than or equal to 1.0, which means the body is shock.

Animals were randomly divided into 3 group (n = 24,each): BC group (blank control group): no shock; NR group (normal resuscitation group): HS + intravenous resuscitation with the return of the shed blood + 2 volumes of normal saline (NS); and DHFZT group: HS + intravenous resuscitation with the return of the shed blood + 2 volumes of NS + DHFZT. 62 mg/kg of DHFZT extract dissolved in 2 ml of 0.9% normal saline was admnistered to DHFZT, but an equal volume of 0.9% saline was given to NR group through jejunum fistula Prepared at before and after the resuscitation and 3, 9 h post-resuscitation. All rats in BC group need perform surgical operations including intubating tube in the right carotid artery and the left femoral artery right and the right femoral vein. Two other groups also need to do so. The animals of NR and DHFZT group were killed at 1, 6, 12 h post-resuscitation. The euthanasia was realized by cervical dislocation. Before rats killed, serum was obtained and stored at −20°C until using for the analysis of endotoxin, IFABP. Small intestine then were harvested for the analysis of p-VASP and ZO-1 protein.

### Measurement of serum endotoxin, IFABP

The serum concentration of endotoxin were measured by EKT-5 M set dynamic Gram-negative bacteria test kit through kinetic turbidimetric assay. The blood samples for IFABP was assayed by commercially available ELISA kits according to the manufacturer’s instructions.

### Analysis of blood flow of the small intestine

To measure blood flow of the small intestine by laser speckle perfusion imaging system.

### Analysis of ZO-1 protein and p-VASP in small intestinal tissue by western blotting and immunohistochemistry

Expression of ZO-1 and p-VASP were measured by Western blotting. Intestinal tissue was frozen, stored at −80°C. Protein (20 μg) was electrophoresed through a 10-20% Tricinegradient gel (GE Healthcare Life Sciences, Beijing), transferred tonitrocellulose membrane (MSI, Westboro) and blocked with TBST (Tris-buffered saline with Tween-20) containing 5% non-fat dry milk. Protein was quantified by a protein nucleic acid analyzer (bicinchoninic acid assay, NanoVue, GE Healthcare Life Sciences, United States). First antibody (1:1000; Santa Cruz Biotechnology) or anti-β-actin (1:2000; Santa Cruz Biotechnology) were incubated overnightat 4°C. Membranes were incubated with horseradish peroxidase-labeled secondary antibody (1:3000, Santa Cruz Biotechnology) and developed with UVP gel imaging analysis analyzer systems (UVP Co, Ltd., United States). Blot densitometrical analyses were performed using the Quantity One software(Bio-Rad Laboratories, Hercules).

In addition, SP immunohistochemistry technique (IHC) was used to evaluate those expression. The tissue sample was fixed in neutral formalin (4%), embedded in paraffin, stained using SP-IHC, DAB color, dried and observed by light microscopy. Immunohistochemical score was measured using the immunoreactive score developed by Remmele and Stagner [15].

### Histopathological observation of intestinal tissue by HE stained

Intestinal tissue was fixed with formaldehyde solution (10%), dehydrated with graded alcohol, embedded in paraffin, sliced into cuts of 4 μm, and stained by hematoxylin eosin staining (HE). Pathological changes of intestinal tissue were observed by light microscopy. Injury of the small intestine were assessed using the score developed by Chiu [16].

### Statistical analysis

Data are presented as the mean ± SD values. SPSS version 17.0 was used to analyse the data. For comparison among groups, T tests and one-way analysis of variance (ANOVA) tests were used. P-value less than 0.05 was considered statistically significant.

## Results

### Change of Mean Arterial Pressure (MAP) and Shock Index (SI)

**Figure 1:**
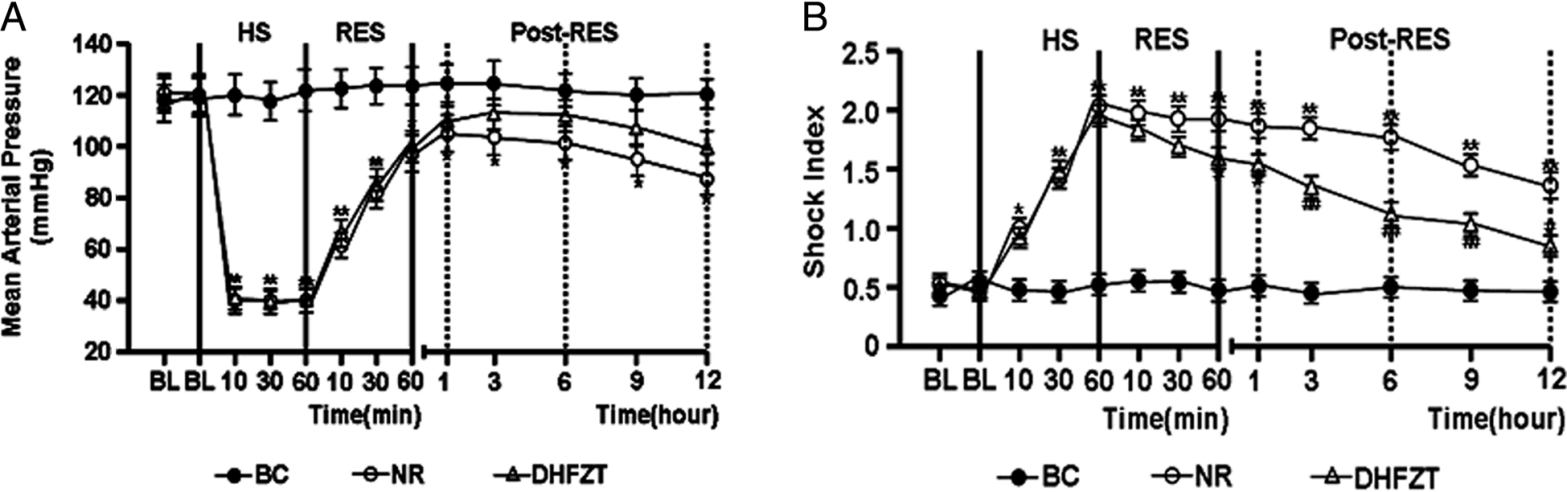
**Change of Mean Arterial Pressure (MAP) and Shock Index (SI). (A)**: change of MAP; **(B)** change of SI. BC group (blank control group) = no shock; NR group (normal resuscitation group) = HS + intravenous resuscitation with the return of the shed blood + 2 volumes of normal saline (NS); DHFZT group (Dai Huang Fu Zi Tang group) = HS + intravenous resuscitation with the return of the shed blood + 2 volumes of NS + DHFZT. BL, baseline;RES, resuscitation. Versus BC, ^*^P<0.05, ^**^P<0.01; Versus NR, ^#^*P*<0.05, ^##^*P*<0.01.

As expected, HS produced and fell to 40 mmHg in MAP in all hemorrhaged rats. There was no significant difference in the level of hypotension between the NR group and DHFZT group. Resuscitation was not fully restored and maintained MAP to pre-HS baseline in NR group and DHFZT group where the shed blood was returned. In contrast, MAP in DHFZT group was only slightly improved at 6, 9, 12 h post-resuscitation compared with NR group, but *P*>0.05 (Figure 1A).To know whether HS model was succesful or not and severity degree of HS, we assessed change of SI in all rats. In Figure 1(B), SI during HS and post-resuscitation in the NR group and DHFZT group was a remarkable increase compared with BC group. After given DHFZT, SI presented a downward trend in post-resuscitation, which indicated that shock status of body was gradually been improved.

### DHFZT significantly increased intestinal blood flow in rats with HS

**Figure 2:**
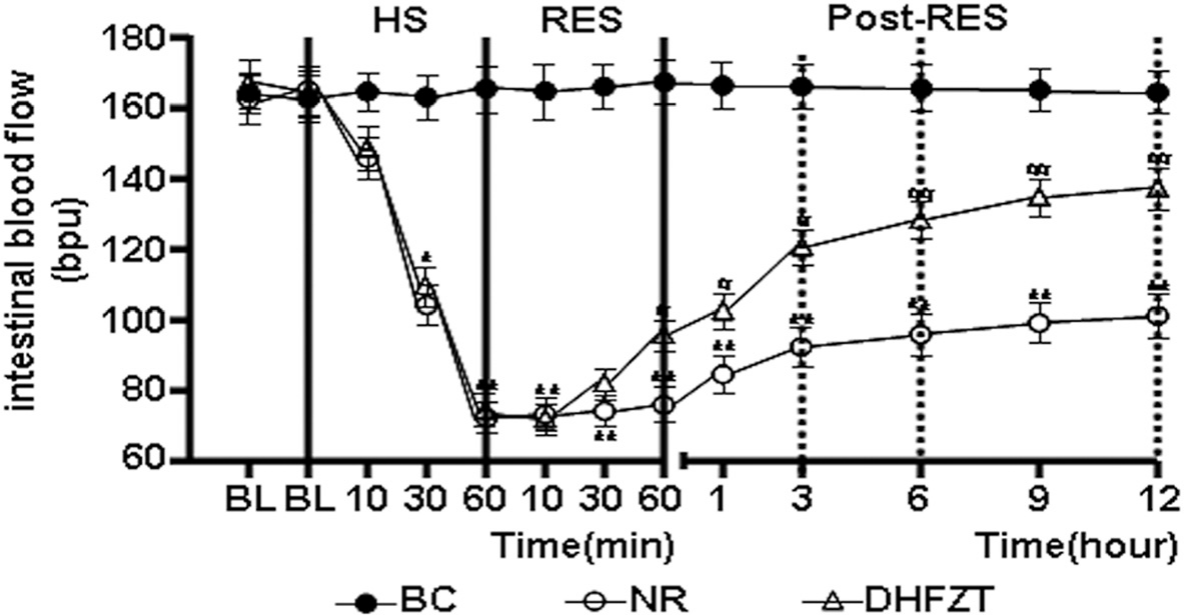
**DHFZT significantly increased intestinal blood flow in rats with HS.** BC group (blank control group) = no shock; NR group (normal resuscitation group) = HS + intravenous resuscitation with the return of the shed blood + 2 volumes of normal saline (NS); DHFZT group(Dai Huang Fu Zi Tang group) = HS + intravenous resuscitation with the return of the shed blood + 2 volumes of NS + DHFZT. BL, baseline; RES, resuscitation; Versus BC, ^*^P<0.05,^ **^P<0.01. Versus NR, ^#^*P*<0.05, ^##^*P*<0.01.

As illustrated in Figure 2, HS caused a marked reduction in intestinal blood flow. In NR group, intestinal blood flow with 60 minutes has not been improved by resuscitaion with the return of the shed blood + 2 volumes of normal saline (NS). Intestinal blood flow after HS has gradually increased slowly after 1 h post-resuscitation, but much less than BC group at post-resuscitation. Compared with NR group, intestinal blood flow in DHFZT group increased significantly since 60 minutes resuscitation until 12 h post-resuscitation (*P* < 0.05 and *P* < 0.01, respectively).

### Effects of DHFZT on ZO-1 and p-VASP in intestinal tissue by western blotting and immunohistochemical staining

**Figure 3:**
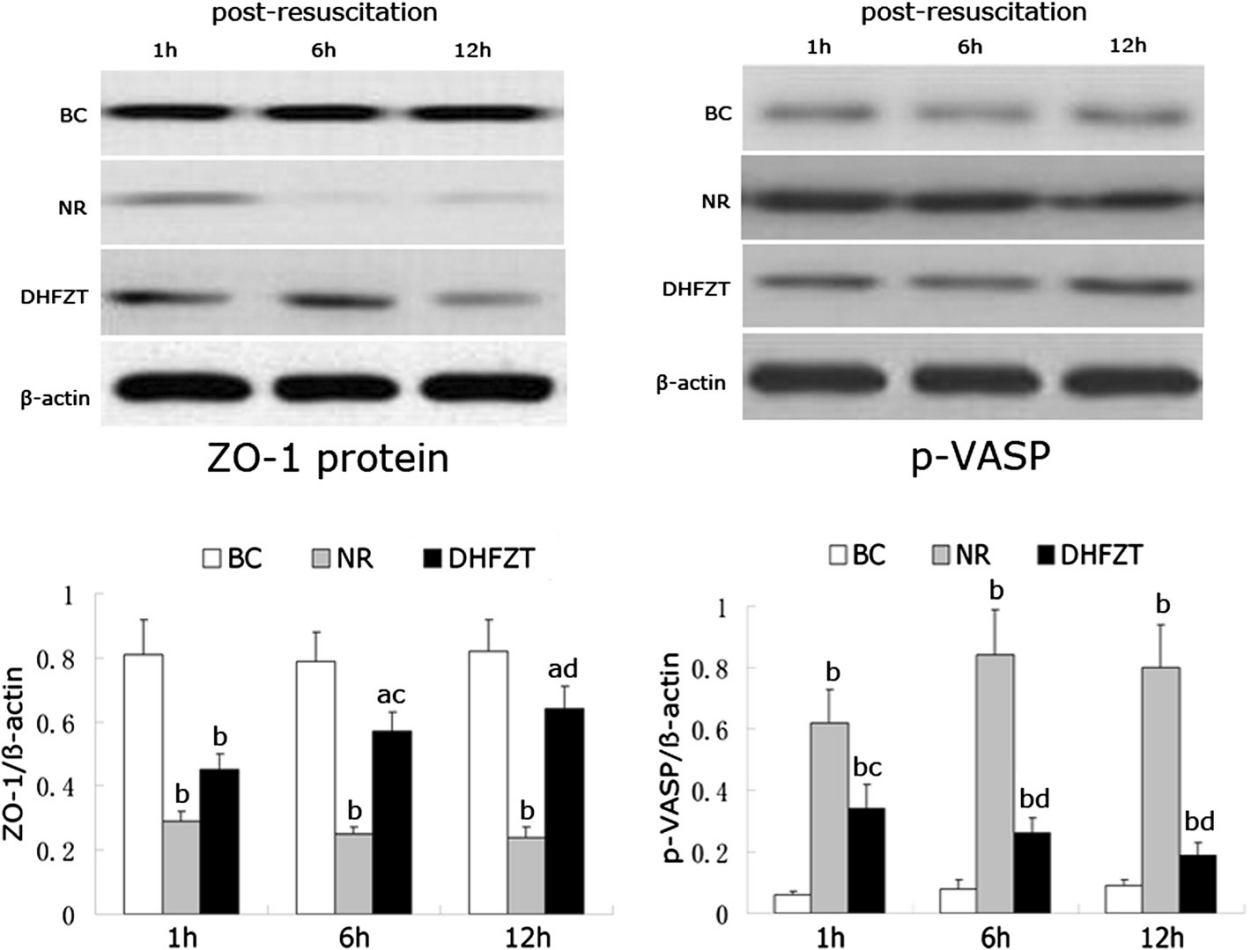
**Effects of DHFZT on ZO-1 and p-VASP protein in intestinal tissue measured by western blotting.** ZO-1, zonula occludens-1; p-VASP, phosphorylated vasodilator-stimulated phosphoprotein; BC, blank control group; NR, normal resuscitation group; DHFZT, Dai Huang Fu Zi Tang group. BL, baseline; RES, resuscitation; β-actin was used as an intrinsic control. Versus BC, ^a^*P*<0.05, ^b^*P*<0.01; Versus NR, ^c^*P*<0.05, ^d^*P*<0.01.

**Figure 4:**
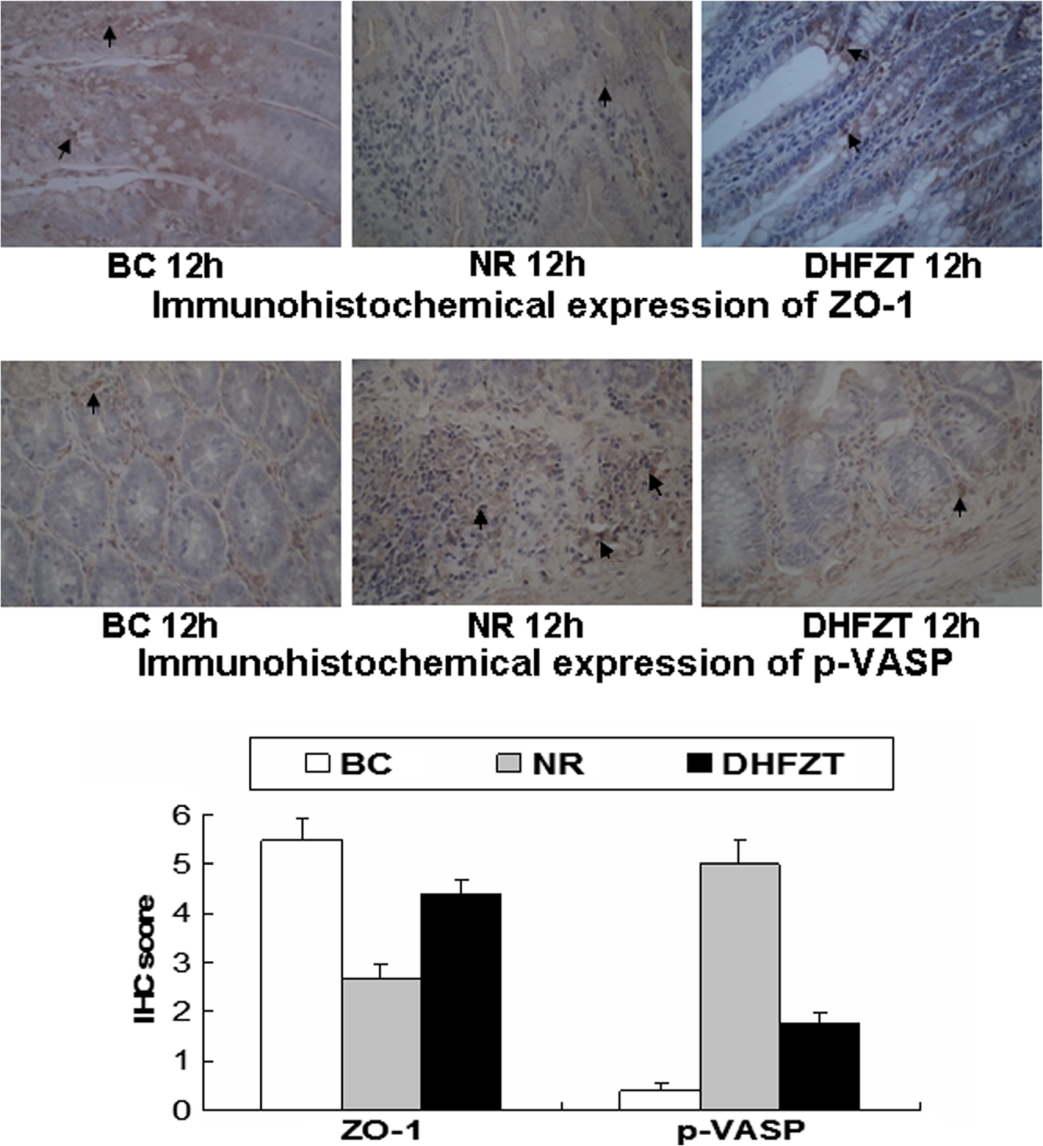
**Effects of DHFZT on ZO-1 and p-VASP protein in the intestinal tissue by immunohistochemical staining at 12 h post-resuscitation.** ZO-1, zonula occludens-1; p-VASP, phosphorylated vasodilator-stimulated phosphoprotein; BC, blank control group; NR, normal resuscitation group; DHFZT, Dai Huang Fu Zi Tang group. Versus BC, ^a^*P*<0.05, ^b^*P*<0.01; Versus NR, ^c^*P* <0.05.

The expression of ZO-1 and p-VASP of intestinal tissue were detected by western blotting at 1, 6, 12 h post-resuscition and immunohistochemical staining at 12 h post-resuscition, as illustrated in Figures 3 and 4. The expression of ZO-1 protein in NR group was significantly downregulated, and p-VASP upregulated by western blotting compared with BC group at 1 h after resuscitation (*P* < 0.01). After DHFZT treatment, the expression of ZO-1 protein was upregulated, and p-VASP downregulated at 6, 12 h post-resuscitation compared with NR group (*P* < 0.05 and *P* < 0.01, respectively). Moreover, immunohistochemical staining was used to illustrate the effect of DHFZT on ZO-1 and p-VASP of intestinal tissue at 12 h post-resuscitation. ZO-1 protein showed a strong positive expression in BC group, and slightly negative expression in NR group and moderate positive expression in DHFZT group at post-resuscitation, but p-VASP was just the opposite. In NR group, The expression of ZO-1 protein was markedly decreased (*P* < 0.01, vs BC group), but p-VASP was substantially increased (*P* < 0.01, vs BC group), which indicated that HS lead to the minute structure damages of the intestinal tissue. Compared with NR group, the expressions of ZO-1 protein was up-regulated at 6, 12 h post-resuscitation(*P* < 0.05 and *P* < 0.01, respectively), but p-VASP down-regulated by DHFZT at 1, 6, 12 h post-resuscitation (1 h, *P* < 0.05; 6, 12 h, *P* < 0.01), indicating the minute structure damages of the intestinal tissue were alleviated by DHFZT after HS.

### DHFZT significantly relieved pathological damage of intestinal tissue and reduced intestinal epithelial damage index

**Figure 5:**
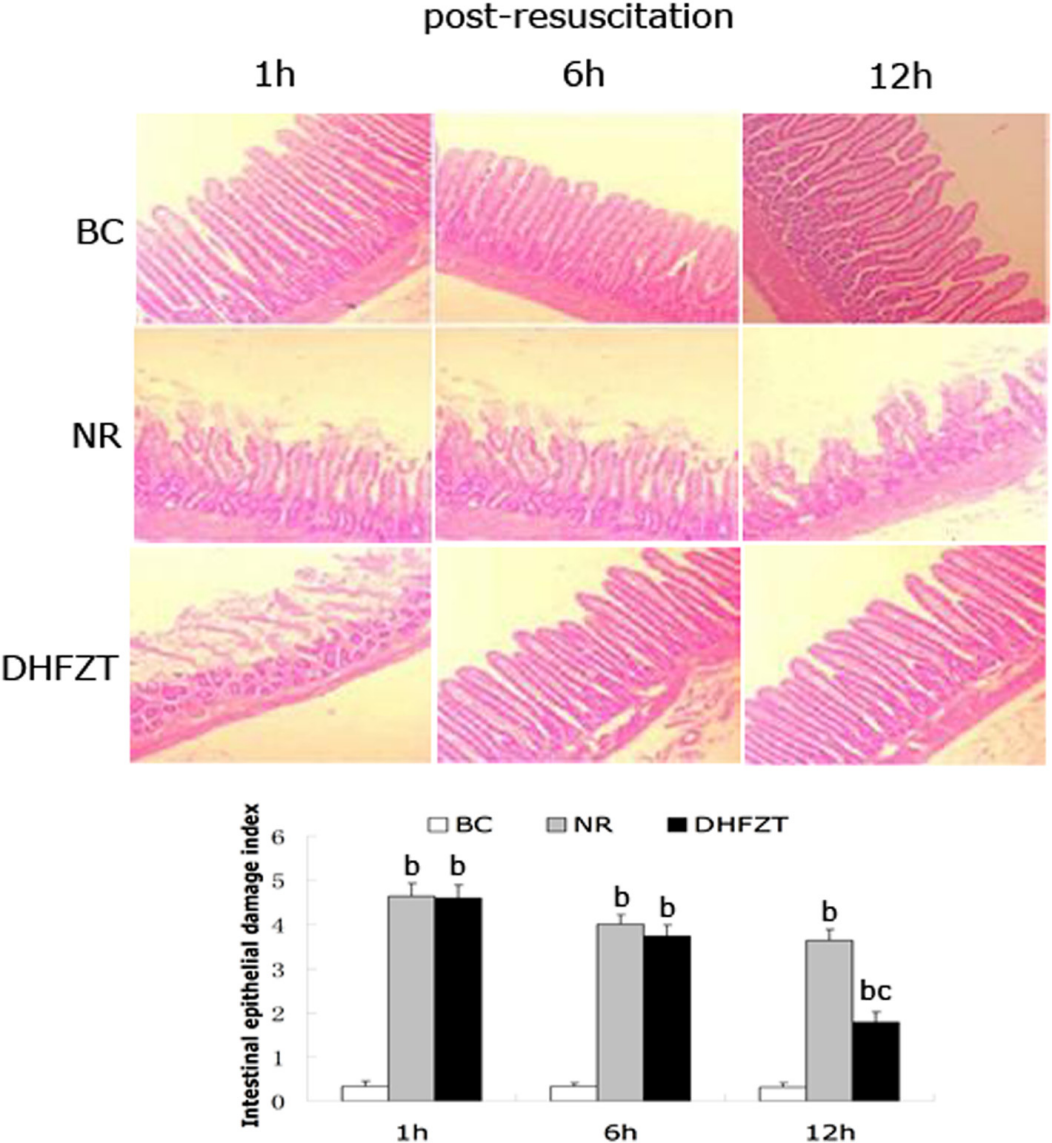
**DHFZT significantly relieved pathological damage of intestinal tissue and reduced intestinal epithelial damage index(magnification, × 100).** HE, hematoxylin-eosin staining; BC, blank control group; NR, normal resuscitation group; DHFZT, Dai Huang Fu Zi Tang group. Versus BC, ^b^*P*<0.01; Versus NR, ^c^*P* <0.05.

The results of HE staining revealed severe edema, massive coloboma, necrosis, shedding, collapsed submucosal vessels, and necrotic mucosal lamina propria glands in NR group. Histologic lesion of intestinal tissue in DHFZT group were significantly reduced at 6, 12 h post-resuscitation compared with NR group. Intestinal epithelial damage index was used to quantitative histological lesions. Intestinal epithelial damage index in NR group was markedly increased after HS compared with BC group (*P* < 0.01). After treatment with DHFZT, intestinal epithelial damage index was significantly decreased at 12 h post-resuscitation compared with NR group (*P* < 0.05), as shown in Figure 5.

### DHFZT significantly reduced the serum concentration of IFABP and endotoxin

**Figure 6:**
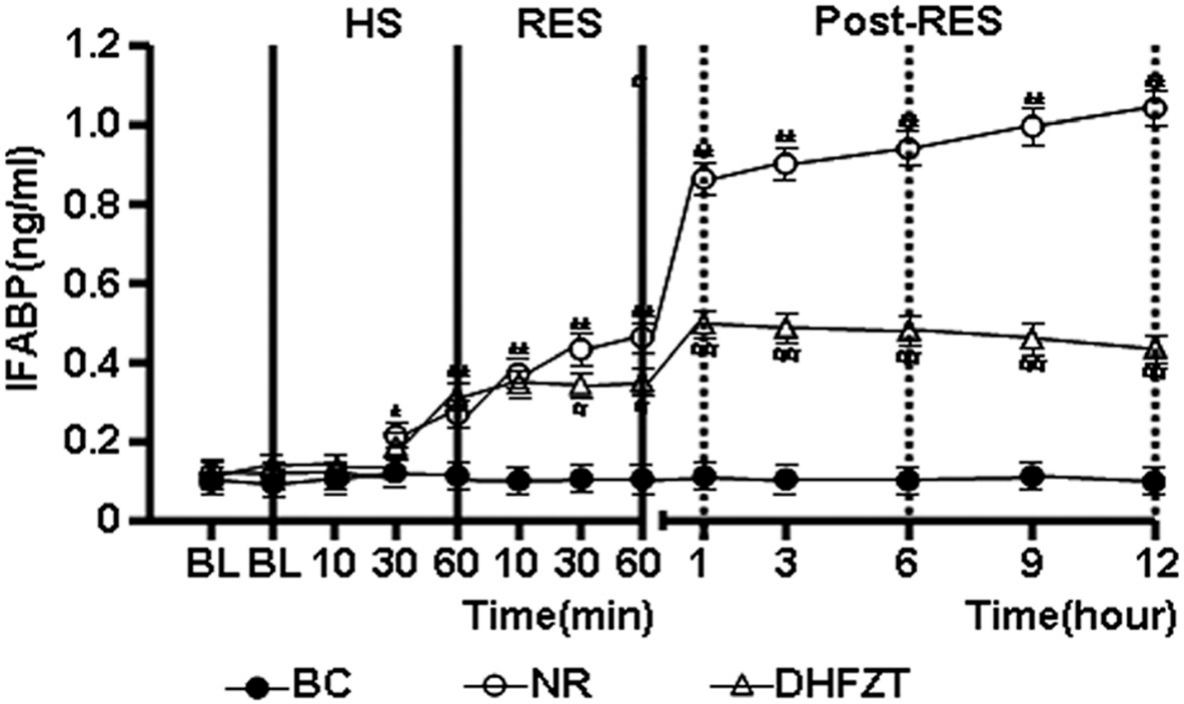
**DHFZT reduce the serum concentration of IFABP.** IFABP, Intestinal fatty acid binding protein; BC, blank control group; NR, normal resuscitation group; DHFZT, Dai Huang Fu Zi Tang group. Versus BC, ^*^P<0.05, ^**^P<0.01, and Versus NR, ^#^*P* <0.05, ^##^*P*<0.01.

**Figure 7:**
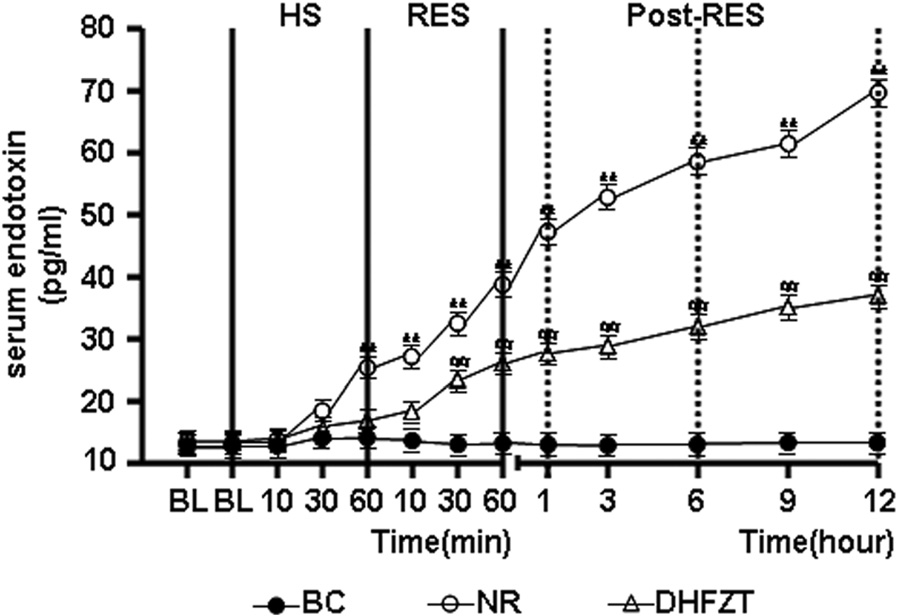
**DHFZT reduce the serum concentration of endotoxin.** BC, blank control group; NR, normal resuscitation group; DHFZT, Dai Huang Fu Zi Tang group. Versus BC, ^*^P<0.05, ^**^P<0.01, and Versus NR, ^#^*P* <0.05, ^##^*P*<0.01.

HS caused a marked elevation in the serum concentration of IFABP and endotoxin. In NR group, the serum concentration of IFABP and endotoxin has not been significantly reduced by resuscitaion with the return of the shed blood + 2 volumes of normal saline (NS) singly at post-resuscitation. But rats in DHFZT group showed significant decrease of endotoxin and IFABP concentration at 30, 60 minute resuscitation (*P* < 0.05) and 1, 3, 6, 9, 12 h (*P* < 0.01) post-resuscitation compared with these of NR group, as illustrated in Figures 6 and 7.

## Discussion

The present study is the first to investigate the effects of DHFZT on intestinal blood blow, expression of p-VASP and ZO-1, and intestinal endotoxaemia after HS. Our studies demonstrate the following: (1) HS causes a marked reduction in intestinal blood flow, and a significant elevation in serum endotoxin, which are not improved by normal resuscitation with shed blood + 2 volumes of NS; (2) The expression of ZO-1 protein is significantly downregulated, but p-VASP upregulated after HS, indicating that HS leads to the minute structure damages of the intestinal tissue; (3) DHFZT earlier restores the intestinal blood flow since 30 minutes resuscitaion until 12 h post-resuscitation, but nou restores MAP.In addition, DHFZT aslo upregulates the expression of ZO-1 protein, and downregulates the expression of p-VASP in intestinal tissue and significantly ameliorates the pathological damage of intestinal tissue. (4) DHFZT can distinctly decrease the serum concentration of endotoxin and reduce the severity of intestinal endotoxaemia.

HS is induced by massive blood loss after trauma or surgery, which leads to severe reduction in circulating blood volume and tissue hypoperfusion. HS is characterized by a series of physiologic compensatory adjustments to maintain the blood-supply of vital organs like heart and brain aimed at saving the life of the organism. At the same time, the blood supply of the intestine is greatly reduced, and with resuscitation, a repersusion injury is introduced to cause obligatory fluid sequestration and enhance intestine-derived endotoxemia and exaggerated systemic inflammatory respose. Given this interrelated pathophysiology, it is reasonable to suggest that intestinal blood blow should be restored as soon as possible. A study has shown that the shock-induced intestinal microvascular derangemengts and endothelial cell dysfunction can only partially be attennated with a resuscitation regimen containing blood [17]. The present study aslo has demonstrated that DHFZT obviously increased intestinal blood blow and serum concentration of IFABP realesed from broken gastrointestinal epithelial cells, which can be easily detected when the intestinal mucosa is ischemia and hypoxia. Serum concentration of IFABP is a marker of damage of the intestinal mucosa [18,19]. The authors found that when HS, IFABP quickly entered into the blood, and then leaded to high serum IFABP, indicating HS causes damage of the intestine [20]. But this has not improved by shed blood and NS. It is interesting that, as shown in this study, DHFZT shows significant decrease of serum concentration of IFABP. The result demostrates that DHFZT have a therapeutic and protective effect on intestinal mucosal injury after HS. The protective effect is testfied that the pathological damage of intestinal tissue in HS is sharply reduced after administration of DHFZT.

The intercellular junction of the intestinal epithelial cells is responsible for maintaining intestinal mechanical barrier [21,22]. Tight junction is regarded as the most important foundation for maintaining the structure of intestinal mucosal mechanical barrier. ZO-1 protein is one of tight joint structural proteins that found in the surface of the cytoplasmic membrane [23]. Its main function is to maintain and regulate the barrier function, which also involves regulation of cell proliferation and differentiation, maintaining epithelial polarity, transporting cell material. VASP, as a protein involved in skeletal cells, belongs to a family of Ena/VASP proteins [24,25]. It is located in the intercellular junction, focal adhesion of stress fiber terminal and is a highly dynamic change area on the cellular membrane. VASP has three phosphorylation sites, two of which are for serine loci (Serl57, Ser239) and the other is for THR loci (Thr278). In normal intestinal epithelial cells (IEC), VASP and ZO-1 are both located in the intercellular junction. VASP in the aspects of expression, distribution density is closely associated with ZO-l protein [26]. Recovery of tight junction and barrier function depend on VASP phosphorylation. VASP can mediate morphological change of ZO-1 by phosphorylating Serl57, which regulates tight junction and cell barrier function. In this study, the expression of p-VASP in the NR group was significantly increased compared with the BC group, but markedly decreased and significantly increased in the DHFZT group, which indicated DHFZT can protect intercellular junction of the intestinal epithelial cells, and then maintain normal intestinal mechanical barrier by regulating the expression of ZO-1 protein and VASP phosphorylation. Luyer et al. found that the tight junction protein ZO - l was lost and p-VASP increased under low intestinal blood flow in shock rats, at the same time, permeability of the intestinal mucosa and bacteria translocation have also increased [27]. The study aslo has showed the more expressions of ZO-1, the more intestinal blood flow in NR group and DHFZT group. But the relationship between p-VASP and intestinal blood flow was just the opposite.

The injury of intestinal mucosa barrier caused by intestinal ischemia could increase permeability of intestinal mucosa for endotoxin, which afterwards give rise to endoxemia and serious consequences such as the systemic inflammatory response syndrome (SIRS), the multiple organ dysfunction syndrome (MODS) etc. Few studies have shown that the prognosis of HS closely relates to the enterogenous endotoxemia, which cannot be reverted by intravenous fluid resuscitation [28,29]. Therefore, reducing the concentration of the enterogenous endotoxin will help to improve the prognosis of HS. The results of our study indicated that the level of serum endotoxin remarkablely elevated since 60 minutes after HS, which indicate that HS gave rise to intestinal dysbacteriosis and the overgrowth of Gram-negative bacteria, then a large amounts of endotoxin permeated the damaged intestinal mucosal barrier and entered the bloodstream.

## Conclusions

The results of our study indicate that DHFZT siginificantly improves intestinal blood flow, protects the intestinal barrier function by up-regulating the expression of ZO-1 protein and down-regulating expression of p-VASP, and finally ameliorates intestinal endotoxaemia after HS. But DHFZT can not markedly improve the reduction of mean arterial pressure in HS. In short, these results suggest that DHFZT may be a useful herbal formula for restoring ingtestinal blood flow, protecting intestinal mechanical barrier and reducing the degree of enterogenous endotoxemia when the HS.

## Abbreviations

HS: Hemorrhagic shock; MAP: Mean arterial pressure; SI: Shock index; DHFZT: Dai Huang Fu Zi Tang; p-VASP: Phosphorylated vasodilator-stimulated phosphoprotein; ZO-1: Zonula occludens-1 protein; IFABP: Intestinal fatty acid binding protein; HE: Hematoxylin eosin staining; IHC: Immunohistochemistry technique.

## Competing interests

All authors are in agree with the content of the manuscript and declare no financial or intellectual competing interests regarding this study.

## Authors’ contributions

Conceived and designed the experiments: XG Lu, X K and LB Zhan. Performed the experiments: X K, CY Lv, ZW Fan, YL W, R Ali, CH Lv, SY Li, JH Mu. All authors read and gave final approval for the version submitted for publication.

## Pre-publication history

The pre-publication history for this paper can be accessed here:

http://www.biomedcentral.com/1472-6882/14/80/prepub
